# Maternal contributions to pregnancy success: from gamete quality to uterine environment

**DOI:** 10.1590/1984-3143-AR2023-0085

**Published:** 2023-09-08

**Authors:** Anna Carolina Denicol, Luiz Gustavo Bruno Siqueira

**Affiliations:** 1 Department of Animal Science, University of California, Davis, CA, United States of America; 2 Embrapa Gado de Leite, Juiz de Fora, MG, Brasil

**Keywords:** oocyte, embryo, assisted reproduction, developmental competence

## Abstract

The establishment and maintenance of a pregnancy that goes to term is *sine qua non* for the long-term sustainability of dairy and beef cattle operations. The oocyte plays a critical role in providing the factors necessary for preimplantation embryonic development. Furthermore, the female, or maternal, environment where oocytes and embryos develop is crucial for the establishment and maintenance of a pregnancy to term. During folliculogenesis, the oocyte must sequentially acquire meiotic and developmental competence, which are the results of a series of molecular events preparing the highly specialized gamete to return to totipotency after fertilization. Given that folliculogenesis is a lengthy process in the cow, the occurrence of disease, metabolic imbalances, heat stress, or other adverse events can make it challenging to maintain oocyte quality. Following fertilization, the newly formed embryo must execute a tightly planned program that includes global DNA remodeling, activation of the embryonic genome, and cell fate decisions to form a blastocyst within a few days and cell divisions. The increasing use of assisted reproductive technologies creates an additional layer of complexity to ensure the highest oocyte and embryo quality given that *in vitro* systems do not faithfully recreate the physiological maternal environment. In this review, we discuss cellular and molecular factors and events known to be crucial for proper oocyte development and maturation, as well as adverse events that may negatively affect the oocyte; and the importance of the uterine environment, including signaling proteins in the maternal-embryonic interactions that ensure proper embryo development. We also discuss the impact of assisted reproductive technologies in oocyte and embryo quality and developmental potential, and considerations when looking into the prospects for developing systems that allow for *in vitro* gametogenesis as a tool for assisted reproduction in cattle.

## Introduction

The ability to successfully establish a pregnancy that goes to term and results in healthy offspring is perhaps the most important aspect of livestock production. Both dairy and beef operations rely upon cows calving at regular intervals to achieve economic and environmental sustainability. Research efforts in the field of reproductive biology have been directed to determine morphological, cellular, and molecular features involved in successful development of a pregnancy, and these efforts have greatly advanced our understanding about the mechanisms underlying oocyte quality and developmental competence, oviduct and uterine environment, and maternal recognition of pregnancy [for excellent reviews on these topics the reader is directed to Hansen and Tribulo (2019); Mathew et al. (2022); Moorey et al. (2022)]. Nevertheless, a considerable proportion (40 to 60%) of pregnancies are still lost in the first few weeks after ovulation (Wiltbank et al., 2016) and the widespread use of Assisted Reproductive Technologies (ART) has brought more attention to these losses.

Gametes and embryos respond to environmental cues and, thus, events occurring before and after ovulation can affect the developmental potential of the oocyte, zygote, and early embryo. Oocyte competence to undergo nuclear and cytoplasmic maturation, be fertilized and become a zygote begins prior to ovulation, when the preovulatory follicle and the oocyte itself must complete a series of cellular events (Blondin et al., 2002). Several processes are required by the oocyte to support early embryonic development: cytoplasmic accumulation of maternal mRNA (Krisher, 2004), modification of organelles (Krisher and Bavister, 1998), and meiotic resumption (Sirard, 2001). The oocyte plays an essential role in supplying the early embryo with mRNAs and organelles through the maternal-to-zygotic transition and beyond; therefore, any alterations in the oocyte cytoplasm during oogenesis and/or maturation may negatively impact the embryo derived from that oocyte. For instance, previous studies have indicated that oocytes matured *in vivo* are more competent to support embryo development compared with their *in vitro*-matured counterparts (Rizos et al., 2002; Krisher, 2004). These differences may be due to gene expression, transcript and protein abundance in the early embryo derived from those oocytes (Vigneault et al., 2004; Banliat et al., 2022).

Similarly, preimplantation embryos are sensitive to the environment and their development may be affected by signaling molecules, either present within the maternal reproductive tract or during *in vitro* artificial culture conditions (Hansen and Tribulo, 2019; Ealy et al., 2021; Wooldridge et al., 2022). It is not surprising that conditions during the preimplantation period may exert short-, mid-, and long-term effects upon the embryo because this relatively short window of development involves a multitude of events to set the stage for future progression of pregnancy. For instance, epigenetic marks such as DNA methylation are lost (global demethylation) and re-inserted (*de novo* methylation) during reprogramming (Santos and Dean, 2004; Burdge and Lillycrop, 2010) under the influence of the preimplantation environment. Pronuclei syngamy, the first cleavage divisions, degradation of maternal mRNA, minor and major embryonic genomic activation (Hamatani et al., 2004), differentiation of the inner cell mass (ICM) that will eventually result in embryonic organogenesis (Ralston and Rossant, 2005; Kojima et al., 2014) and differentiation of extraembryonic tissues (placentation) (Wang and Dey, 2006; Grazul-Bilska et al., 2010), are amongst the distinct events occurring during preimplantation that may be affected by environmental signals.

Finally, it is well documented that alterations in the peri-ovulatory microenvironment and later on during preimplantation embryonic development can have long-term effects on the fetus during pregnancy and, ultimately, offspring function and health after birth (Rivera, 2019; Siqueira et al., 2019). Less understood is the impact of events occurring earlier in folliculogenesis, i.e., during the preantral and early antral stages, and to what extent these events might influence oocyte quality and developmental competence. This review article explores different aspects of follicle growth, oocyte quality, and maternal environment for early embryo development that could impact the likelihood of successful pregnancy establishment in cattle. Recent advances in the understanding of the putative mechanisms involved in developmental alterations, as well as the prospect of *in vitro* gametogenesis, will also be discussed.

## Oocyte quality

Oocyte quality is intrinsically related to follicle development (Eppig et al., 2002), and therefore maintaining oocyte viability throughout the long process of folliculogenesis is critical to ensure proper embryo development and ultimately pregnancy success. During the growth phase, the oocyte must sequentially acquire meiotic competence (i.e., the ability to leave the prophase I arrest and proceed to metaphase II in response to the LH surge – meiotic competence is acquired during the preantral to early antral transition) and developmental competence, or the ability to support embryo development after fertilization (acquired during antral follicle development) [reviewed by Schultz et al. (2018)]. At approximately 65-70% of their final volume, oocytes have accumulated about 95% of total and poly-A RNA that will be present in the fully-grown oocyte (Sternlicht and Schultz, 1981). Transcriptomic analysis of mouse oocytes isolated from primordial, primary, secondary, small antral and large antral follicles revealed a complex and highly regulated sequence of events, including upregulation of genes and networks involved in cell cycle regulation, protein synthesis, DNA replication, and others (Pan et al., 2005). Interestingly, the group with most differential pattern of gene expression was the one of oocytes from primordial follicles, demonstrating that 1) these oocytes are transcriptionally active; and 2) the primordial to primary transition is a critical step for successful oogenesis and folliculogenesis (Pan et al., 2005).

A detailed understanding of the requirements and determinants of oocyte quality during preantral folliculogenesis (which spans the resting primordial follicle up to the late or multi-layer secondary follicle, leading up to antrum formation) is still missing in large species such as cattle. However, studies examining the long-term effects of altered physiological states on oocyte quality and fertility demonstrate that the importance of a healthy environment for oocyte growth extends much beyond the antral follicle wave. It has been demonstrated in cattle that the occurrence of severe negative energy balance or disease (reproductive or otherwise) during the early postpartum period affects the quality of the ovulatory oocyte up to 60 days later (Garverick et al., 2013; Ribeiro et al., 2016; Marei et al., 2022). Culture of murine preantral follicles at the early secondary stage (starting follicle diameter of 100-130 µm) in the presence of non-esterified fatty acids altered follicular development as evidenced by lower rate of antrum formation, changes in gene expression and steroidogenic profile. Although follicles and oocytes developed under these conditions, cleavage and development of the resulting embryos to the blastocyst stage was impaired (Valckx et al., 2014).

Association studies in cattle confirm that severe negative energy balance during the transition period affects fertility; a recent study demonstrated several differences in the transcriptomic profile of pre-ovulatory follicle granulosa cells at eight weeks post-partum when they originated from cows with high or low concentration of non-esterified fatty acid in blood at two weeks post-partum (Marei et al., 2022). Although a reliable *in vitro* system that allows the growth of bovine preantral follicles to the pre-ovulatory stage and beyond is not yet available, short-term follicle or ovarian cortex culture have unraveled inflammatory processes, oxidative stress and apoptosis among the factors potentially involved in impairment of oocyte viability (Leroy et al., 2005; Marei et al., 2019; Pedroza et al., 2022). Similarly, *in vitro* culture of bovine ovarian cortex in the presence of lipopolysaccharides (LPS) increased the production of pro-inflammatory cytokines and induced markers of primordial follicle activation (Bromfield and Sheldon, 2013).

The external environment can also have a long-term negative impact on oocyte quality. Perhaps the best example is that of heat stress and the dramatic detrimental effect it has on cattle fertility. The detrimental effects of heat stress are multifactorial; oocyte quality and hormonal production take weeks to months to return to physiologic levels after exposure to summer heat [reviewed by Roth (2015)], indicating that preantral follicles are being affected. The molecular reasons for this long-term impact in preantral follicles and oocytes have been investigated to some extent *in vitro* and include loss of follicle growth and viability, in addition to upregulation of transcripts related to apoptosis and stress response. However, results have been inconsistent and variable according to stage of preantral follicle examined, whether follicles were enclosed in the ovarian stroma or isolated before culture, and the regimen of heat stress applied (Paes et al., 2016; Aguiar et al., 2020).

A recent study examined the transcriptomic profile of single bovine oocytes between 60 and 120 µm in diameter and found that the oocyte undergoes significant changes in gene expression as it progresses through the growth phase (Latorraca et al., 2023). Although the follicle size was not specifically measured in that study, the initial oocyte diameter likely corresponds to a secondary stage preantral follicle or a follicle that is transitioning into the antral phase. This study indicates that the oocyte is transcriptionally active and undergoing significant changes as it develops, confirming the findings from murine oocytes. Although not yet published for bovine follicles, it has been demonstrated in the mouse that the oocyte undergoes extensive changes in DNA methylation during the growth phase [reviewed by Anckaert and Fair (2015)]. This agrees with the idea of the massive genome reorganization that the highly specialized oocyte must undergo in order to support embryo development.

The influence of hormones on preantral follicle and oocyte development has been a topic of investigation for many years. The gonadotropin follicle stimulating hormone (FSH) has been implicated in the regulation of preantral follicle growth, however *in vitro* results have been somewhat inconclusive. Although *in vivo* studies in large animals have been scarce, it has been demonstrated that hypophysectomized ewes had impaired preantral follicle development, with the population of primary follicles being the most affected. The FSH receptor is found in follicles starting at the primordial or primary stages of development of several mammalian species [reviewed by Morton et al. (2023 in press)] and bovine follicles at the primary stage of development respond to FSH by increasing the levels of cAMP (unpublished observations). Supplementation of culture medium with FSH accelerates bovine follicle development, although not dramatically (Candelaria et al., 2020). Accordingly, a recent study in mice demonstrated that successive ovarian stimulations for oocyte recovery resulted in accelerated depletion of the preantral follicle reserve (Wang et al., 2022). Although this has not yet been examined in cattle, this study is relevant given the still prevalent use of hormonal stimulation before oocyte collection for *in vitro* embryo production, particularly in *Bos taurus* breeds in North America, where the United States is one of the top producers of *in vitro* cattle embryos worldwide.

In summary, folliculogenesis and oogenesis of cattle are lengthy processes that are subjected to the effects of the internal as well as the external environment. Due to the difficulty of studying preantral folliculogenesis and the absence of efficient culture systems, most of the knowledge available today comes from rodents, with cattle studies being more limited to association in vivo studies and short-term in vitro culture. The refinement of less invasive techniques to allow the retrieval of preantral follicles from the ovaries of living cows, associated with optimization of *in vitro* culture conditions, will help shed light into the mechanisms regulating oocyte development and the factors determining oocyte competence for pregnancy success.

## Maternal environment

### Epigenetics and signaling pathways

Early embryo development involves a series of molecular events including the so-called epigenetic reprogramming (Niemann et al., 2010). This biological phenomenon begins at the zygote stage and is responsible for making embryonic cells totipotent due to global genome demethylation, with the exception of imprinted genes. Later, embryonic cells must undergo *de novo* methylation before differentiation of specific cell types. Reprogramming of epigenetic marks such as DNA and histone methylation is controlled by DNA methyltransferases, histone methyltransferases, and histone acetyltransferases or deacetylases (Inbar-Feigenberg et al., 2013). The preimplantation period of development is also marked by the first cell-fate decision of the embryo, characterized by differentiation of polarized and depolarized blastomeres into ICM or trophectoderm (TE). In mice, first cell-fate decision is directed by expression of *NANOG* by the inner embryo cells (ICM) or *CDX2* by the outer TE cells through the *Hippo* signaling pathway (Leung and Zernicka-Goetz, 2013).

In the specific case of ART, particularly *in vitro* embryo production (IVP), it is noteworthy that all these events occur during *in vitro* culture. Considering the fact that artificial culture systems are very distinct from the natural maternal reproductive tract and the early embryo is sensitive to environmental cues, suboptimal conditions may and will affect further embryonic development and pregnancy success. The mechanisms are still not fully understood, but evidence support the idea that disruptions of epigenetic marks might be involved in the differences between *in vivo* and *in vitro* embryos (Hiendleder et al., 2006; Katari et al., 2009). In that regard, there is strong evidence that alterations in gene expression patterns resultant from changes to the epigenome are a major cause of abnormalities subsequent from a disturbed environment such as *in vitro* culture (Calle et al., 2012; Feuer et al., 2014), including imprinting disorders (Fernández-Gonzalez et al., 2004; Horsthemke and Ludwig, 2005; El Hajj and Haaf, 2013; Urrego et al., 2014) and abnormal expression of non-imprinted genes (Katari et al., 2009). Epigenetic modifications have been observed following IVP in humans and cattle (Katari et al., 2009; Chen et al., 2013) and are characterized by loss of methylation in imprinted genes in those species (Doherty et al., 2000; Market-Velker et al., 2010; Chen et al., 2015).

Previous studies have evaluated DNA methylation in bovine fetuses with a phenotype characterized by overgrowth, referred to as ‘large offspring syndrome’. Affected fetuses presented biallelic expression of several imprinted genes due to loss of methylation in one of the alleles in different fetal tissues (Chen et al., 2013). Loss of methylation has also been detected by RNAseq analysis of allele specific expression of imprinted genes (Chen et al., 2015). From a developmental perspective, *in vitro* embryo production systems disrupt distinct epigenetic features of the embryonic cells and may exert negative effects on further fetal development.

### Molecular oviductal and uterine features during preimplantation

The oviductal and uterine environments are an intricate network of substances and signaling molecules that may act directly or indirectly on the embryo to affect embryonic development and subsequent pregnancy (Wolf et al., 2003; Halter et al., 2011; Lonergan and Forde, 2014). The distinct environments can program evolutionary adaptations required for species survival through advantageous adaptations to life after birth. Animal models have been used to demonstrate the effects of an altered maternal environment upon cellular and molecular characteristics of the embryo. In rats, a low-protein diet fed to pregnant dams induced changes in insulin, glucose and amino acids concentrations. Those alterations in maternal serum metabolites and uterine microenvironment led to alterations in embryonic cell numbers apparently as a result of compromised rate of cellular proliferation. Blastocysts had decreased numbers of TE and ICM cells compared with embryos collected from dams fed a normal diet (Kwong et al., 2000). Physiologically, the uterus is a nutrient-rich environment that is the sole source to support early preimplantation embryo development. The uterine metabolome during the first seven days of the estrous cycle has been characterized in the cow. Numerous putative unique metabolites (147) have been identified and included metabolites that have signaling capacities and the ability to affect embryo development. Moreover, the uterine metabolome changed over time, from ovulation until day 7 of the cycle, evidence of the dynamic nature of the maternal environment (Tribulo et al., 2019).

In addition, assessment of gene expression patterns between ovulation and day 7 of the estrous cycle has also been used to identify potential molecules that could affect embryo development during the preimplantation period in the bovine uterus and oviduct (Tribulo et al., 2018). A total of 93 target genes were studied and all were expressed in the reproductive tract; those included hormones, growth factors, chemokines, cytokines and signaling molecules. A time-dependent expression pattern was observed, with some genes regulated by estradiol during early phases of the cycle and other set of genes regulated by progesterone as the cycle progressed. These so-called ‘embryokines’ identified as maternally-derived signaling molecules with potential effects to regulate early embryo development and, therefore, a putative role to improve outcomes of *in vitro* embryo production if added to culture medium.

## Assisted reproductive technologies

### Advantages and limitations

The benefits of ART in the field of animal breeding to promote rapid progress in genetic gain are well recognized in several domestic animals and considered very important, particularly for livestock production. The past years have witnessed a consistent increase in the use of ART to produce high-genetic merit animals, with a total reported number of ~1.9 million cattle embryos transferred globally in 2021 (Viana, 2022). The global number of *in vivo* derived (MOET) embryos has been superior than the number of IVP embryos since the beginning of embryo data collection by the International Embryo Technology Society (IETS) in the early 1980´s; however, a shift in these numbers occurred in 2017 and the production and transfers of IVP embryos was greater than MOET embryos for the first time on record (Viana, 2018). This shift was not surprising because a trend of increasing numbers of IVP and a stagnated/decreasing numbers of MOET embryos has been observed for several years by the IETS data retrieval committee. In livestock, particularly cattle, the use of ART has been continuously increasing, mostly to produce genetically superior animals. Of the 1.9 million embryos produced in 2021, approximately 80% were IVP (Viana, 2022).

Among the main potential advantages of IVP over MOET that have made it such a popular technique are 1) the dramatic reduction in generation interval due to oocyte retrieval from pre-pubertal females (not feasible with MOET), and 2) the acceleration in the spread of elite genetics since OPU can be performed more frequently than MOET and oocytes can be retrieved from donors up to the end of the first trimester of pregnancy, i.e., IVP is more suitable for large-scale embryo transfers programs due to its ‘economy of scale’ feature. When incorporated into the routine reproductive management in dairy farms, embryo technologies have also been successfully used to bypass low fertility during summer heat-stress in high producing lactating dairy cows, with an important advantage in terms of pregnancy rates compared with artificial insemination (Stewart et al., 2011; Vasconcelos et al., 2011; Baruselli et al., 2020).

Despite the apparent success in the adoption and use of ART (embryo technologies) by dairy and beef operations, there is still some challenges and bottlenecks involved, particularly for IVP embryos. Evidence indicates that disruptions in biological process during oocyte *in vitro* maturation, fertilization, and embryo culture probably caused by the artificial environment can lead to abnormal development with potential lifetime impacts on the offspring (Fleming et al., 2015; Siqueira et al., 2019). The quality of immature cumulus-oocyte complexes aspirated by ovum pick-up (OPU) are also affected by disturbances in final follicular growth, because oocyte competence to support embryo development relies on accumulation of maternal transcripts in their cytoplasm.

As a consequence of these artificial conditions during critical phases of oocyte/zygote/embryo development, blastocysts produced *in vitro* differ from their naturally derived (*in vivo*) counterparts both in morphology and molecularly (Rinaudo and Schultz, 2004). Conversion of cleaved zygotes into blastocysts is fairly low (Thompson, 1997; Lazzari et al., 2002; Lonergan et al., 2006), gene expression (Enright et al., 2000; Giritharan et al., 2007; McHughes et al., 2009) and epigenetic marks (Fernández-Gonzalez et al., 2004; Urrego et al., 2014) may be disrupted, and the actual blastocyst is exposed to metabolic stress by *in vitro* conditions (Gardner and Harvey, 2015).

Practical consequences of the putative disturbances to developmental processes caused by an artificial environment have implications on the outcomes after the transfer of an IVP embryo. In cattle, the risk of a successful pregnancy is reduced if an IVP embryos is transferred into a recipient compared with *in vivo*-derived embryos (Hasler, 2000; Pontes et al., 2009; Siqueira et al., 2009; Ferraz et al., 2016). If pregnancy establishment occurs, early and late embryonic losses are more frequent in dams carrying an IVP embryo/fetus (Pohler et al., 2016) and the chance of abortions is increased (Farin and Farin, 1995; Wang et al., 2004). Fetal morphology and development can be affected (Bertolini et al., 2002) and changes in placenta molecular features have also been observed for IVP-embryo pregnancies (Salilew-Wondim et al., 2013).

There is substantial evidence that characteristics of offspring from IVP at birth can be different compared with their naturally-conceived counterparts in cattle, sheep, mice and humans (Farin and Farin, 1995; Young et al., 1998; Sinclair et al., 1999; Blondin et al., 2000; Bertolini et al., 2002; Farin et al., 2006; Bloise et al., 2014; Feuer et al., 2014; Lafontaine et al., 2023). Particularly in ruminants, the most common aberrant phenotype in newborns derived by IVP is fetal overgrowth, an abnormal condition frequently referred to as “large offspring syndrome” (Behboodi et al., 1995; Young et al., 1998; Farin et al., 2010). The bovine large offspring syndrome is characterized not only by elevated birth weight, which is possibly the least important problem associated with IVP. There is also increased incidence of abortions, congenital malformations and aberrant perinatal abnormalities (Holm and Callesen, 1998; van Wagtendonk-de Leeuw et al., 1998). The severity of the symptoms, however, may vary from case to case (Farin et al., 2006). Interestingly, adult cattle can also be affected. Cardiomegaly at 13 months of age observed in calves that were heavier at birth (oversized) as a result of IVP (McEvoy et al., 1998). Performance of adult IVP-derived offspring might also be offset, if genetic potential is considered. Results of recent retrospective studies have raised some important questions about lifetime productive and fertility performance in dairy cows (Siqueira et al., 2017; Lafontaine et al., 2023).

### Signaling molecules to mimic maternal reproductive tract

Optimization of *in vitro* procedures and culture systems may enhance blastocyst production and quality, ultimately leading to a greater success in pregnancy and birth of healthy offspring. Therefore, efforts have been made to mimic the natural maternal environment (oviductal and uterine) and the dynamics of changes in the content of these systems. Although much progress has been made, *in vitro* systems are still remarkably different from the maternal environment and the artificial media used for IVP are probably deficient in several regulatory molecules that play important roles in regulating development *in vivo* (Gardner and Harvey, 2015*)*. For instance, surface tension, cell-to-cell interactions, stiffness, salts, sugars, ions, amino acids, miRNAs, signaling molecules, exosomes, and other components may be deficient or even absent in the culture system.

Considering the major differences between *in vivo* and *in vitro* produced embryos, it is reasonable to hypothesize that the conditions in the oviduct and endometrium during early developmental stages enhance developmental competence of embryos. Therefore, attempts to replicate the maternal environment include the addition of growth factors and molecules naturally secreted by the female reproductive tract to media used for IVP (Paria and Dey, 1990; Kane et al., 1997). From the molecular perspective, it has been reported that addition of maternally-derived regulatory molecules to *in vitro* embryo culture medium can alter the DNA methylome in fetal tissue at the end of the third trimester of pregnancy (Li et al., 2020).

In this context, several regulatory molecules have been added to culture systems to improve embryo development to the blastocyst stage. Previous studies have evaluated the effects of different growth factors such as IGF1 (Palma et al., 1997; Sirisathien and Brackett, 2003; Block et al., 2007), EGF (Paria and Dey, 1990; Sirisathien et al., 2003; Arat et al., 2016), HGF, activin A, CTGF, TDGF1 (Kannampuzha-Francis et al., 2017), TGFA and TGFB (Paria and Dey, 1990), and FGF2 (Fields et al., 2011). A positive effect on embryonic development has been reported by the majority of these studies and currently many commercial IVP laboratories have incorporated growth factors in their media composition. Some factors and molecules secreted by the maternal oviduct and uterine environment, embryokines, can act directly upon the embryo and regulate its development (Hansen et al., 2014). The combination of growth factors and maternally-derived molecules also seems to benefit development and survival of IVP embryos, as demonstrated by the use of a proprietary, serum-free culture medium for bovine embryo production (Block et al., 2010).

Pregnancy outcomes may also be improved after the transfer of embryos exposed to regulatory molecules during *in vitro* culture. For instance, treatment with the embryokine CSF2 increased pregnancy rates of IVP embryos (Loureiro et al., 2009; Denicol et al., 2014). Similarly, IVP embryos treated with another embryokine (DDK1) were also more likely to establish a successful pregnancy compared with non-treated embryos (Denicol et al., 2014). Because determinants of pregnancy success are multifactorial, some studies have not observed benefits of treating embryos during culture or, in some cases, the embryo culture system and media composition affected pregnancy outcomes. For example, Amaral et al. (2022) observed a positive effect on development to the blastocyst stage in embryos exposed to serum or serum replacer during culture, but a lower pregnancy rate after the transfer of these embryos into recipients. Moreover, if CSF2 or DKK1 were added to culture medium, actions of theses embryokines were dependent on the presence of serum in the medium. Pregnancy rate was reduced for embryos cultured with no serum and exposed to CSF2 or DKK1, but these molecules did not affect pregnancy if serum was present. Finally, CSF2 actually abolish the negative effect of serum upon pregnancy rate after embryo transfer.

### Potential novel markers of embryo competence

Determining markers of embryo competence to establish a pregnancy has been the focus of much research as it has the potential to improve the efficiency of ART by enabling the early prediction of which embryos are more likely to survive to term. Recently, Rabaglino et al. (2023) combined the integration of transcriptomic datasets with the use of machine learning to predict embryo competence and survival. The authors reported a subset of eight genes, namely *GSTO1, CHSY1, TPI1, YWHAG, CCNA2, LSM4, CDK7,* and *EIF4A3*, which predicted with high accuracy the competence of embryos in different datasets, indicating that these genes might be important biomarkers of embryo competence.

## Reproduction in a dish

### Advances and bottlenecks related to in vitro gametogenesis

The advances in *in vitro* gametogenesis that the field of reproductive biology has seen in the last decade have changed the reality an opened up the potential for dramatic changes in ART. With the field of stem cell biology expanding in agriculturally-relevant species, the development of technologies to enable *in vitro* gametogenesis could make it possible to move into *in vitro* breeding (Goszczynski et al., 2019), exponentially accelerating genetic gain and decreasing the generation interval. Although this has been proposed in cattle (Goszczynski et al., 2019), it could conceptually be applied to any agricultural species as long as there are pluripotent stem cells available.

Although female *in vitro* gametogenesis has been demonstrated in mice for the first time in 2016 (Hikabe et al., 2016), the efficiency of obtaining oocytes, embryos and offspring is still extremely low. This triggered a detailed evaluation of the molecular characteristics of the different cell types created using the system, with comparisons with cells at the same stage obtained *in vivo* (Aizawa et al., 2023 in press). It was found that not the differentiation of pluripotent stem cells into primordial germ cell-like cells, but the subsequent steps of oocyte and follicle development, seemed to be the most critically altered by the *in vitro* system. Most notably, the authors found that the *in vitro*-derived oocytes lacked markers of acquisition of developmental competence and displayed epigenetic alterations such as DNA methylation abnormalities.

Clearly, there is still a lot to learn about the processes leading to the development of a competent oocyte; moreover, although they represent a groundbreaking advance in the ability to make oocytes in a dish, the existing protocols fail to faithfully recapitulate the highly complex, highly regulated process of oogenesis.

## Final remarks

**Figure 1:**
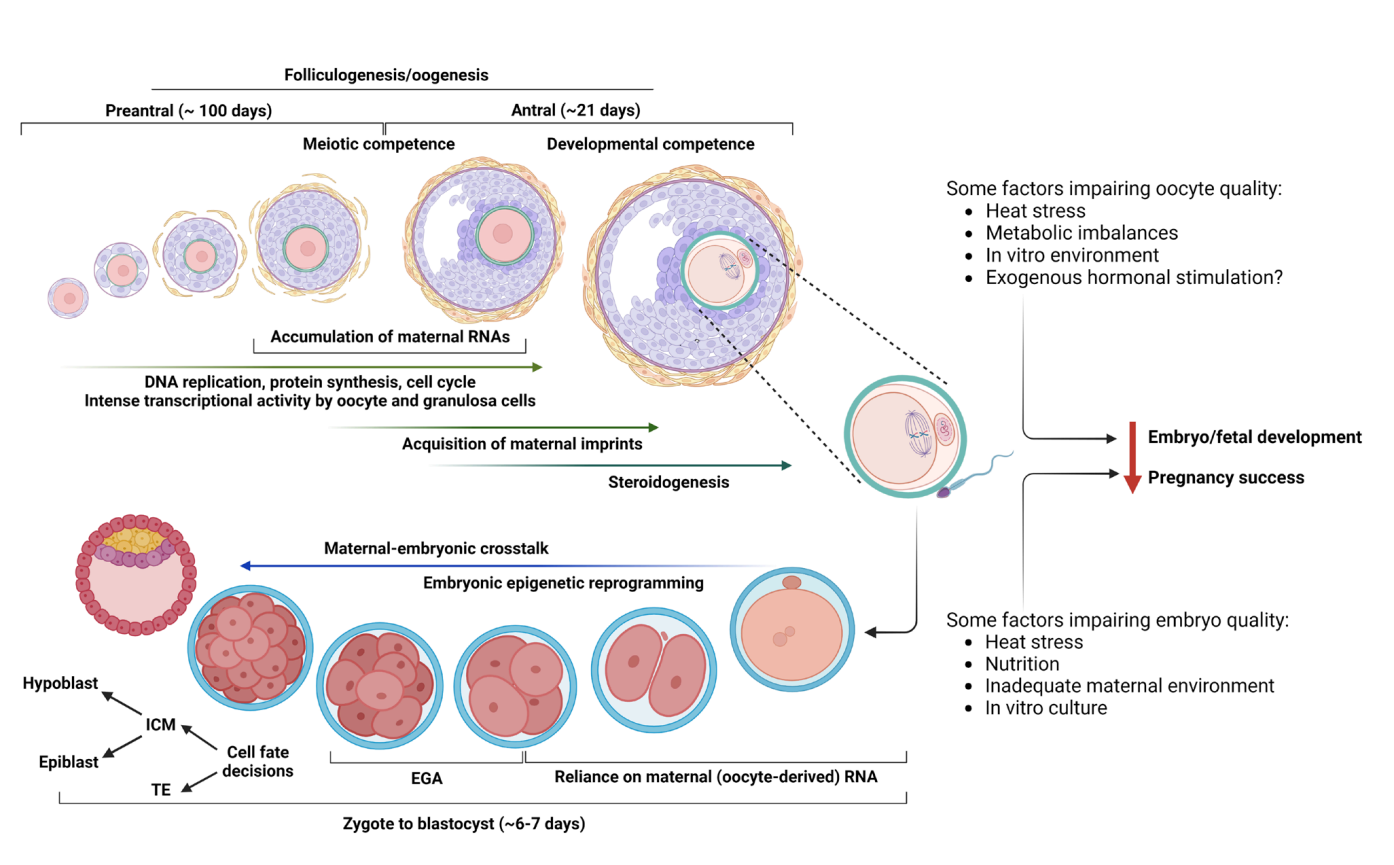
Follicular and embryonic events that are critical for a successful pregnancy. Oocytes are “stored” in the ovary enclosed in a layer of flat granulosa cells in the primordial follicles. Activation and growth of follicles through preantral stages takes several weeks to months, and during this time there is intense activity within the follicular unit with DNA replication, protein synthesis and transcriptional activity. Acquisition of meiotic competence by the oocyte occurs during the transition from late preantral to the early antral phase. Most of the transcripts that the oocyte will synthesize for later embryo use will be stored by the early to mid-antral stage. At this point the oocyte must also acquire developmental competence. Following fertilization of the mature oocyte by sperm, the embryo must now use the resources provided by the oocyte to ensure successful development to the blastocyst stage. Global epigenetic remodeling (with exception of imprinted genes) will be critical to reset the cellular program into a totipotent state. The embryonic genome activation (EGA) that occurs between the 4- and 16-cell stage is critical for the embryo to become transcriptionally autonomous and proceed to the first and second cell fate decisions at the blastocyst stage. There are many internal (e.g. metabolic state) and external (heat stress, in vitro culture) factors that can interfere with one or multiple events and disrupt oocyte and/or embryo development, resulting in failure to establish or maintain a pregnancy. Abbreviations: ICM: inner cell mass; TE: trophectoderm.

The natural microenvironment within the maternal reproductive tract has evolved in mammals to provide full support and program the development of early embryos and fetuses until pregnancy goes to term. Any perturbations to these well-balanced conditions have the potential to affect pregnancy success (establishment and completeness) and offspring health after birth (Figure 1). It is now well recognized that developmental programming starts as early as the initial stages of oocyte growth inside a given ovarian follicle, passing through the period of preimplantation embryonic development, and going up to postnatal life. Thus, the consequences of errors in programming can have lifetime effects upon the offspring.

As the adoption of ART by livestock production systems, particularly IVP, has increased substantially in the past decades, the effects of an altered maternal environment (artificial or non-physiological) have brought great attention to the concepts of programming, especially if one considers the one health concept applied to animal production systems. Different strategies have been developed to overcome the main limitations and bottlenecks of ART, including efforts to preserve/enhance oocyte quality, improvements in culture conditions by addition of maternal-borne factors and, lately, advanced *in vitro* culture systems with one potential future goal being *in vitro* breeding. These technological advances in assisted reproduction will undoubtedly contribute for future livestock productivity and sustainability by accelerating genetic progress with the use of breeding techniques that better mimic the physiological environment and, thus, are more efficient.
